# No difference in effects of ‘PACE steps to success’ palliative care program for nursing home residents with and without dementia: a pre-planned subgroup analysis of the seven-country PACE trial

**DOI:** 10.1186/s12904-021-00734-1

**Published:** 2021-03-07

**Authors:** Rose Miranda, Tinne Smets, Nele Van Den Noortgate, Jenny T. van der Steen, Luc Deliens, Sheila Payne, Katarzyna Szczerbińska, Sophie Pautex, Liesbeth Van Humbeeck, Giovanni Gambassi, Marika Kylänen, Lieve Van den Block, on behalf of PACE, Yuliana Gatsolaeva, Lara Pivodic, Elisabeth Honinx, Marc Tanghe, Hein van Hout, Katherine Froggatt, Bregje Onwuteaka-Philipsen, H. Roeline W. Pasman, Ruth Piers, Ilona Baranska, Mariska Oosterveld-Vlug, Anne B. Wichmann, Yvonne Engels, Myrra Vernooij-Dassen, Jo Hockley, Suvi Leppäaho, Catherine Bassal, Federica Mammarella, Martina Mercuri, Paola Rossi, Ivan Segat, Agata Stodolska, Eddy Adang, Paula Andreasen, Outi Kuitunen-Kaija, Teija Hammar, Rauha Heikkilä, Danni Collingridge Moore, Violetta Kijowska, Maud ten Koppel, Emilie Morgan de Paula

**Affiliations:** 1 Vrije Universiteit Brussel & Ghent University, End-of-Life Care Research Group, Laarbeeklaan 103, 1090 Brussels, Belgium; 2 Vrije Universiteit Brussel, Department of Family Medicine and Chronic Care, Brussels, Belgium; 3 Department of Geriatric Medicine, Ghent University Hospital, Ghent, Belgium; 4 Department of Public Health and Primary Care, Leiden University Medical Center, Leiden, The Netherlands; 5 Department of Public and Occupational Health, Amsterdam Public Health Research Institute, Amsterdam UMC-VU University Medical Center, Amsterdam, The Netherlands; 6 Department of Public Health and Primary Care, Ghent University, Ghent, Belgium; 7 International Observatory on End-of-Life Care, Lancaster University, Lancaster, UK; 8 Laboratory for Research on Aging Society, Department of Sociology of Medicine, Epidemiology and Preventive Medicine Chair, Faculty of Medicine, Jagiellonian University Medical College, Krakow, Poland; 9 Hôpitaux Universitaires de Genève, University of Geneva, Geneva, Switzerland; 10 Department of Internal Medicine, Istituto di Medicina Interna e Geriatria, Università Cattolica del Sacro Cuore, Rome, Italy; 11 National Institute for Health and Welfare, Helsinki, Finland

**Keywords:** Nursing home care, End of life care, Bereavement, Pain, Communication, Neurological conditions

## Abstract

**Background:**

‘PACE Steps to Success’ is a multicomponent training program aiming to integrate generalist and non-disease-specific palliative care in nursing homes. This program did not improve residents’ comfort in the last week of life, but it appeared to improve quality of care and dying in their last month of life. Because this program included only three dementia-specific elements, its effects might differ depending on the presence or stage of dementia. We aimed to investigate whether the program effects differ between residents with advanced, non-advanced, and no dementia.

**Methods:**

Pre-planned subgroup analysis of the PACE cluster-randomized controlled trial in 78 nursing homes in seven European countries. Participants included residents who died in the previous 4 months. The nursing home staff or general practitioner assessed the presence of dementia; severity was determined using two highly-discriminatory staff-reported instruments. Using after-death questionnaires, staff assessed comfort in the last week of life (Comfort Assessment in Dying–End-of-Life in Dementia-scale; primary outcome) and quality of care and dying in the last month of life (Quality of Dying in Long-Term Care scale; secondary outcome).

**Results:**

At baseline, we included 177 residents with advanced dementia, 126 with non-advanced dementia and 156 without dementia. Post-intervention, respectively in the control and the intervention group, we included 136 and 104 residents with advanced dementia, 167 and 110 with non-advanced dementia and 157 and 137 without dementia. We found no subgroup differences on comfort in the last week of life, comparing advanced versus without dementia (baseline-adjusted mean sub-group difference 2.1; *p*-value = 0.177), non-advanced versus without dementia (2.7; *p* = 0.092), and advanced versus non-advanced dementia (− 0.6; *p* = 0.698); or on quality of care and dying in the last month of life, comparing advanced and without dementia (− 0.6; *p* = 0.741), non-advanced and without dementia (− 1.5; *p* = 0.428), and advanced and non-advanced dementia (0.9; *p* = 0.632).

**Conclusions:**

The lack of subgroup difference suggests that while the program did not improve comfort in dying residents with or without dementia, it appeared to equally improve quality of care and dying in the last month of life for residents with dementia (regardless of the stage) and those without dementia. A generalist and non-disease-specific palliative care program, such as PACE Steps to Success, is a useful starting point for future palliative care improvement in nursing homes, but to effectively improve residents’ comfort, this program needs further development.

**Trial registration:**

ISRCTN, ISRCTN14741671. Registered 8 July 2015 – Retrospectively registered.

**Supplementary Information:**

The online version contains supplementary material available at 10.1186/s12904-021-00734-1.

## Background

Between 14 and 29% of people aged 65 years and over in many developed countries die in nursing homes [1]. However, the quality of dying and end-of-life care in this setting, even in countries with high levels of palliative care development, is sub-optimal [2, 3]. To contribute high-quality evidence to address this problem, we developed ‘PACE Steps to Success’, which is a multicomponent program aiming to integrate generalist and non-disease-specific palliative care into nursing homes in six steps using a train-the-trainer approach. Nursing home staff are trained to deliver high-quality palliative care to all residents, from advance care planning to care up to and beyond death [4]. Between 2015 and 2017, we evaluated this program in a seven-country cluster-randomized controlled trial (RCT). The primary trial analyses showed that ‘PACE Steps to Success’ did not improve the comfort in the last week of life (primary outcome) in the overall nursing home population, but it appeared to improve quality of care and dying in the last month of life for this population, although the latter was the secondary outcome [5].

Because this program was designed for all residents and included only three dementia-specific elements [4], we hypothesized that its effects might differ between those with and without dementia in favor of those with mild/moderate or no dementia compared with advanced dementia. People with dementia, especially those with advanced dementia, have wide-ranging physical, cognitive and behavioral impairments, which make their palliative care needs distinct from those without dementia [6, 7]. It is often assumed that for palliative care programs to be effective for people with dementia, they should specifically address the needs of this population [7–9]. Therefore, at the outset of the trial, we planned a subgroup analysis using the same outcome measures as in the primary trial analyses to test this hypothesis [10]. Understanding whether the program effects differ between people with and without dementia while taking dementia severity into account could inform future development of palliative care programs for nursing home residents, of whom between 60 and 83% die with dementia [11]. The present study aims to answer the research question: “Do the effects of the PACE Steps to Success program on comfort in the last week of life and quality of care and dying in the last month of life differ between residents with advanced, non-advanced and without dementia?”

## Methods

This is a pre-planned subgroup analysis of the PACE cluster randomized controlled trial (see data analyses plan submitted as an official deliverable to the European Commission in Additional file 1) [4, 5, 10]. This cluster-RCT was conducted in 78 nursing homes in Belgium, England, Finland, Italy, the Netherlands, Poland and Switzerland to compare PACE Steps to Success with usual care (2015–2017). This trial was registered at http://www.isrctn.com on July 30, 2015 (ISRCTN14741671). Randomization was performed at the nursing home level as the program involved the training of nursing home staff. After baseline measurement, randomization was stratified by country and median number of beds in a 1:1 ratio. Randomization was blinded and performed by independent statisticians. Because of the nature of the study, blinding of treatment was not possible for researchers or participants. More details about the PACE cluster-RCT have been published elsewhere [4, 5]. We reported this study following the CONSORT guidelines for randomized trials.

### Program description

PACE Steps to Success was implemented over the course of 1 year, including 2 months for preparation, 6 months training for nursing home staff in the six steps, and 4 months consolidation. All countries had one or more country trainers. Each nursing home assigned one to six staff members as PACE coordinators. After being trained by two experienced trainers, the country trainers trained and supported the PACE coordinators who were in turn responsible for training and supporting fellow staff. The six PACE Steps included: 1) advance care planning with residents and families; 2) assessment, care planning, and review of resident needs and problems; 3) coordination of care via monthly multidisciplinary palliative care review meetings; 4) high-quality palliative care with a focus on pain and depression; 5) care in the last days of life and 6) care after death [4]. The program included three dementia-specific elements: communication training in advanced dementia for the PACE coordinators, and two elements integrated into the training for all nursing home staff which emphasized dementia as a terminal illness (as part of Step 2) and offered symptom control strategies for residents with and without dementia (in Step 4) [4, 5].

### Participating nursing homes

From a list of nursing homes, those located in a predefined country-specific geographical location were approached randomly by telephone or e-mail to invite them to participate in the study and to evaluate eligibility criteria using a standardized checklist. Inclusion criteria were the provision of on-site nursing care and personal assistance with activities of daily living and off-site medical care by general practitioners (GPs), having at least 30 beds, 15 or more residents having died in or outside the nursing home in the previous year to obtain sufficient power, consent to participation from management in writing before randomization, and agreement to allocate approximately 0.5 days per week for staff to act as PACE coordinators. We excluded nursing homes that had pilot-tested the program materials or used detailed palliative care guidelines/planning tools, the Gold Standards Framework and InterRAI-PC [4, 5].

### Data collection and respondents

One contact person per nursing home identified all residents who had died in the previous 4 months. After-death structured questionnaires for each resident were sent to the staff member most involved in care (preferably a nurse), nursing home administrator and GP at baseline (month 0) and post-intervention (months 13 and 17). As sensitivity analyses showed no difference between program effects using the two post-intervention data, these combined post-intervention data were used in the primary analyses [5]. In this subgroup analysis, we included residents for whom the presence and severity of dementia was determined, classified into three subgroups: advanced, non-advanced and without dementia. We deviated from our pre-planned subgroups (residents with and without dementia), so that we could better investigate the difference between residents with advanced and without dementia.

### Measurements and outcomes

Nursing home staff and GP reported whether a resident “had dementia” or “was diagnosed with dementia”. Dementia was considered present if at least one indicated it was and not present when both indicated it was not or when one indicated this but the other neither returned the questionnaire nor answered the question. Dementia severity was determined using two highly-discriminatory staff-reported instruments, Cognitive Performance Scale (CPS) and Global Deterioration Scale (GDS); those with CPS scores of 5–6 and GDS stage 7 were classified as having advanced dementia, the others as non-advanced dementia. CPS classifies residents into six hierarchical cognitive performance categories, with higher scores indicating worse cognitive impairment [12]. GDS stage 7 indicates very severe cognitive and functional deterioration [13].

Nursing home administrators reported a resident’s sex and age at time of death. Staff assessed functional status 1 month before death in terms of dependency level with eating, dressing and mobility using the Bedford Alzheimer Nursing Severity-Scale: categorized into ‘independent’, ‘needs assistance’, or ‘fully dependent’ [14].

Primary outcome was staff-reported comfort in the last week of life using the validated Comfort Assessment in Dying–End-of-Life in Dementia (CAD-EOLD) scale; see comprehensive description of outcomes in Additional file 2 [15, 16]. CAD-EOLD comprises four subscales: physical distress, dying symptoms, emotional distress and well-being. The CAD-EOLD total scores range between 14 and 42, with higher scores indicating better comfort. CAD-EOLD was found to have better psychometric properties and user-friendliness than other comfort measures in a mixed nursing home population, including residents with and without dementia [17–19]. Secondary outcome was staff-reported quality of care and dying in the last month of life measured using the validated Quality of Dying in Long Term Care (QOD-LTC) scale, comprising ‘personhood’, ‘preparatory tasks’ and ‘closure’ subscales [20]. The QOD-LTC total scores range between 11 and 55, with higher scores indicating better quality of care and dying.

### Statistical analyses

Linear mixed models were used to analyze continuous outcomes and account for the clustered nature of data, with staff, nursing home and country as random factors (only random intercepts) and group (intervention versus usual care), time (post-intervention combining data collected at months 13 and 17 versus baseline) and their interaction as fixed factors. We analyzed differential effects by calculating differences in mean change (post-intervention combining data collected at months 13 and 17 minus baseline) between the subgroups, both for the intervention and control groups (interaction group*time*dementia). For the differential effects, we present estimated differences (and 95% Confidence Intervals) in mean change between the subgroups. All hypothesis testing was two-sided. *P*-values and 95% Confidence Intervals were not adjusted for multiple testing. To address multiplicity concerns with Bonferroni correction, *p*-values should be compared against a 1% significance level to address multiplicity concerns examining dementia subgroups [21]. In individual subgroups, we presented estimated mean scores and mean differences between groups post-intervention. All analyses were on an intention-to-treat and a complete-case basis, assuming data were missing at random. All statistical analyses were conducted using SAS 9.4 software (©SAS Institute Inc., USA).

## Results

**Fig. 1:**
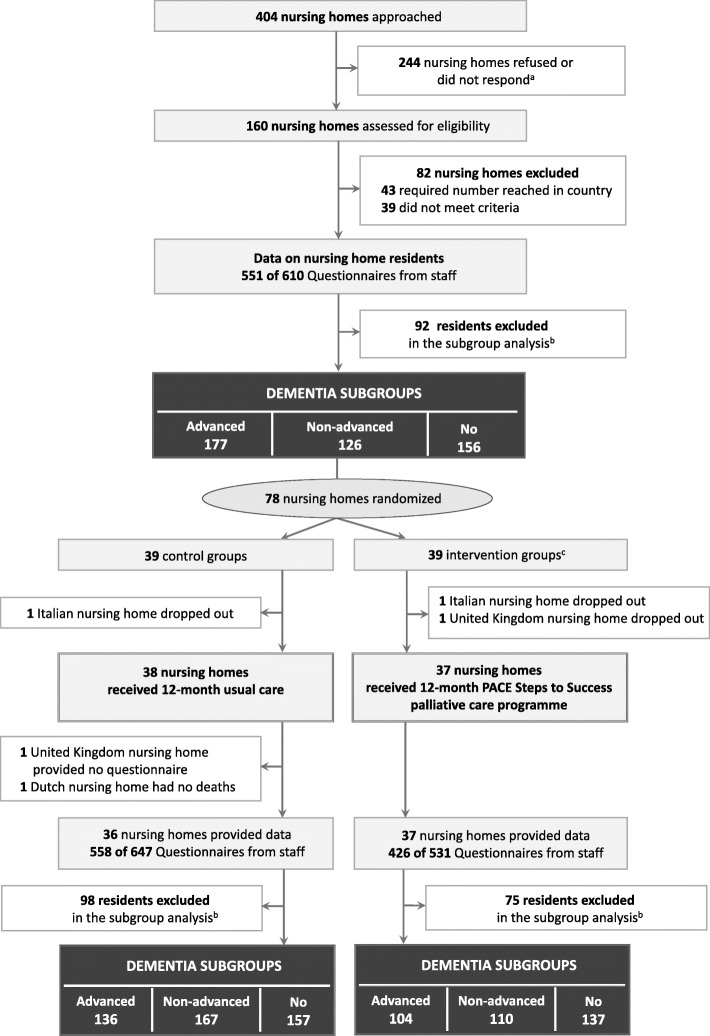
Flowchart of the identification of subgroups at baseline and post-intervention. ^a^ Reasons for refusal included insufficient time, no interest, understaffing, already involved in other studies, change in management. ^b^ Excluded in the subgroup analysis, because the presence and severity of dementia could not be determined. ^c^ Pre-implementation phase (months 1–2), implementation phase (months 3–8), and consolidation phase (months 9–12)

Of the 160 nursing homes assessed for eligibility, 82 were excluded (43 were excluded as the required number of nursing homes were reached in the country and 39 did not meet the inclusion criteria) (Fig. 1). Of the 78 nursing homes randomized, 1 nursing home in the control group and 2 nursing homes in the intervention group dropped out. Between the program implementation and the post-intervention measurements, 2 nursing homes in the control group dropped out. At baseline, we included 177 residents with advanced dementia, 126 with non-advanced dementia and 156 without dementia (Fig. 1). In the control group post-intervention, we included 136 residents with advanced dementia, 167 with non-advanced dementia and 157 without dementia. In the intervention group post-intervention, we included 104 residents with advanced dementia, 110 with non-advanced dementia and 137 without dementia. We excluded 92 residents at baseline and 98 (control group) and 75 (intervention group) residents post-intervention, as the presence and severity of dementia could not be determined.

Table 1 provides a detailed description of the characteristics of the residents in the three subgroups for the baseline and the post-intervention measurements. At time of death, average age was between 82.5 and 87.5 years at baseline and between 84.0 and 86.9 years post-intervention. They were predominantly female, with percentages ranging from 53.7 to 70.7% at baseline and from 56.1 to 67.7% post-intervention. Between 73.3 and 97.8% of residents with advanced dementia were ADL-(activities of daily living) dependent for eating, dressing and mobility. Among those with non-advanced and no dementia, between 42.4 and 71.4% were ADL-dependent for dressing and mobility, while between 19.1 and 32.8% were fully dependent for eating.

**Table 1:** Characteristics of residents by subgroups

TOTAL SAMPLE FOR THE SUBGROUP ANALYSIS	Baseline scores (T0)		Post-intervention (T1 + T2)	
	Control	Intervention	Control	Intervention
	*N* = 238	*N* = 221	*N* = 460	*N* = 351
**Advanced dementia**	**(*n*** **= 99)**	**(*n*** **= 78)**	**(*n*** **= 136)**	**(*n*** **= 104)**
**Age at time of death**, unadjusted mean (SD)	85.5 (7.3)	86.3 (8.6)	86.1 (8.0)	86.5 (8.3)
**Gender**, female, unadjusted frequency, n (%)	70 (70.7)	47 (60.3)	88 (64.7)	64 (61.5)
**Eating dependency**^**a**^**,** n (%)				
- Independent	0 (0)	1 (1.3)	0 (0)	3 (2.9)
- Needs assistance	17 (17.3)	18 (23.4)	26 (19.1)	21 (20.4)
- Fully dependent	81 (82.7)	58 (73.3)	110 (80.9)	79 (76.7)
**Dressing dependency**^**a**^**,** n (%)				
- Independent	0 (0)	0 (0)	0 (0)	0 (0)
- Needs assistance	6 (6.1)	4 (5.2)	3 (2.2)	7 (6.7)
- Fully dependent	92 (93.4)	73 (94.8)	133 (97.8)	97 (93.3)
**Mobility dependency**^**a**^**,** n (%)				
- Independent	1 (1.0)	2 (2.6)	2 (1.5)	2 (1.9)
- Needs assistance	12 (12.4)	9 (11.7)	11 (8.3)	16 (15.5)
- Fully dependent	84 (86.6)	66 (85.7)	120 (90.2)	85 (82.5)
**Non-advanced dementia**	**(*n*** **= 65)**	**(*n*** **= 61)**	**(*n*** **= 167)**	**(*n*** **= 110)**
**Age at time of death**, unadjusted mean (SD)	87.1 (7.9)	87.5 (7.5)	85.7 (7.8)	86.9 (6.1)
**Gender**, female, unadjusted frequency, n (%)	47 (57.7)	36 (59.0)	113 (67.7)	73 (66.4)
**Eating dependency**^**a**^**,** n (%)				
- Independent	6 (9.4)	13 (21.7)	33 (20.1)	28 (25.5)
- Needs assistance	37 (57.8)	30 (50.0)	97 (59.1)	61 (55.5)
- Fully dependent	21 (32.8)	17 (28.3)	34 (20.7)	21 (19.1)
**Dressing dependency**^**a**^**,** n (%)				
- Independent	1 (1.6)	3 (5.2)	8 (4.8)	6 (5.6)
- Needs assistance	17 (27.0)	19 (32.8)	58 (34.9)	41 (38.0)
- Fully dependent	45 (71.4)	36 (62.1)	100 (60.2)	61 (56.5)
**Mobility dependency**^**a**^**,** n (%)				
- Independent	13 (20.3)	6 (10.2)	29 (17.5)	14 (13.0)
- Needs assistance	18 (28.1)	28 (47.5)	57 (34.3)	39 (36.1)
- Fully dependent	33 (51.6)	25 (42.4)	80 (48.2)	55 (50.9)
**Without dementia**	**(*n*** **= 74)**	**(*n*** **= 82)**	**(*n*** **= 157)**	**(*n*** **= 137)**
**Age at time of death**, unadjusted mean (SD)	82.5 (12.2)	83.2 (9.6)	84.0 (10.9)	84.2 (10.2)
**Gender**, female, unadjusted frequency, n (%)	50 (67.6)	44 (53.7)	88 (56.1)	79 (57.7)
**Eating dependency**^**a**^**,** n (%)				
- Independent	18 (25.0)	25 (31.6)	55 (35.5)	47 (35.6)
- Needs assistance	34 (47.2)	33 (41.8)	68 (43.9)	51 (38.6)
- Fully dependent	20 (27.8)	21 (26.6)	32 (20.6)	34 (25.8)
**Dressing dependency**^**a**^**,** n (%)				
- Independent	3 (4.2)	11 (13.8)	14 (9.0)	21 (6.1)
- Needs assistance	25 (34.7)	25 (31.3)	63 (40.6)	103 (29.9)
- Fully dependent	44 (61.1)	44 (55.0)	78 (50.3)	220 (64.0)
**Mobility dependency**^**a**^**,** n (%)				
- Independent	4 (5.5)	14 (18.2)	22 (14.2)	25 (18.9)
- Needs assistance	29 (39.7)	24 (31.2)	55 (35.5)	42 (31.8)
- Fully dependent	40 (54.8)	39 (50.6)	78 (50.3)	65 (49.2)

Missing cases – Advanced dementia, baseline: age = 3; gender = 4; BANS-S = 3 | post-intervention measurements: age = 6; gender = 3; BANS-S = 3). Non-advanced dementia, baseline: age = 6; gender = 5; BANS-S = 3 | post-intervention measurements: age = 6; gender = 6; BANS-S = 2). Without dementia, baseline: age = 3; gender = 4; BANS-S = 7 | post-intervention measurements: age = 12; gender = 8; BANS-S = 7

*Abbreviations*: *SD* standard deviation, *BANS-S* Bedford Alzheimer Nursing Severity-Scale

^a^ Measured using BANS-S one month before death (range 7–28). Higher scores indicate greater severity. Unadjusted frequencies

The program effects on comfort in the last week of life did not differ statistically between residents with advanced and without dementia (subgroup differences in baseline-adjusted mean differences 2.1; 95% CI − 0.9–5.1; *p* = .177), those with non-advanced and without dementia (2.7; − 0.4–5.9; *p* = .092), or those with advanced and non-advanced dementia (− 0.6; − 3.8–2.5; *p* = .698) (Table 2). The baseline-adjusted mean differences in comfort scores were − 1.9 without dementia to 0.8 with non-advanced dementia (Table 3).

**Table 2:** Effects on comfort and quality of care and dying by subgroups

Comparison between the subgroups	Subgroup differences in baseline-adjusted mean difference (95% CI)	***p***-values^**c**^
**Comfort in the last week of life**^**a**^		
Advanced dementia **vs** Without dementia	2.1 (−0.9–5.1)	0.177
Non-advanced dementia **vs** Without dementia	2.7 (−0.4–5.9)	0.092
Advanced dementia **vs** Non-advanced dementia	−0.6 (−3.8–2.5)	0.698
**Quality of care and dying in the last month of life**^b^		
Advanced dementia **vs** Without dementia	−0.6 (−4.1–2.9)	0.741
Non-advanced dementia **vs** Without dementia	−1.5 (−5.2–2.2)	0.428
Advanced dementia **vs** Non-advanced dementia	0.9 (−2.8–4.6)	0.632

All mean total scores and *p*-values are cluster-adjusted

*Abbreviations*: *CAD-EOLD* Comfort Assessment in Dying–End of Life in Dementia, *QOD-LTC* Quality of Dying in Long Term Care, *CI* confidence intervals

^a^ Comfort in the last week of life was measured using CAD-EOLD scale (total scores range 14–42). Higher scores indicate better comfort

^b^ Quality of care and dying in the last month of life was measured using QOD-LTC scale (total scores range 11–55). Higher scores indicate better quality of care and dying

^c^ Subgroup differences in the estimated baseline-adjusted mean differences between intervention and control groups post-intervention (group x time x dementia interaction)

**Table 3:** Cluster-adjusted mean scores and differences by subgroups

Individual subgroups	Baseline scores (T0)				Post-intervention scores (T1 + T2) ^d^				Baseline-adjusted mean difference intervention versus control group post-intervention
	Control		Intervention		Control		Intervention		
	Cases No.	Mean^b^	Cases No.	Mean^b^	Cases No.	Mean^b^	Cases No.	Mean^b^	
**Comfort in the last week of life**^**a**^									
Advanced dementia	***n*** **= 99**		***n*** **= 78**		***n*** **= 136**		***n*** **= 104**		0.2
	91	30.6	74	30.8	131	30.3	97	30.7	
Non-advanced dementia	***n*** **= 65**		***n*** **= 61**		***n*** **= 167**		***n*** **= 110**		0.8
	60	30.0	57	30.0	157	31.0	102	31.8	
Without dementia	***n*** **= 74**		***n*** **= 82**		***n*** **= 157**		***n*** **= 137**		−1.9
	70	29.7	75	30.6	146	31.3	128	30.2	
**Quality of care and dying in the last month of life**^b^									
Advanced dementia	***n*** **= 99**		***n*** **= 78**		***n*** **= 136**		***n*** **= 104**		3.6
	97	38.1	75	37.1	135	38.0	103	40.6	
Non-advanced dementia	***n*** **= 65**		***n*** **= 61**		***n*** **= 167**		***n*** **= 110**		2.7
	65	38.4	59	38.3	163	39.5	104	42.2	
Without dementia	***n*** **= 74**		***n*** **= 82**		***n*** **= 157**		***n*** **= 137**		4.2
	74	41.2	78	39.3	152	39.8	133	42.2	

All mean differences between groups post-intervention are cluster and baseline adjusted

*Abbreviations*: *CAD-EOLD* Comfort Assessment in Dying – End of Life in Dementia, *QOD-LTC* Quality of Dying in Long Term Care

^a^ Comfort in the last week of life was measured using CAD-EOLD scale (total scores range 14–42). Higher scores indicate better quality of dying

^b^ Quality of care and dying in the last month of life was measured using QOD-LTC scale (total scores range 11–55). Higher scores indicate better quality of end-of-life care

^c^ Total scores are averages per subscale multiplied by total number of items. Cases with missing data on more than 50% of items per subscale were excluded from the calculation of the total scale scores

^d^ Post intervention measurements collected for residents at T1 (=month 13) and T2 (=month 17)

The program effects on quality of care and dying in the last month of life also did not differ statistically between advanced and no dementia (− 0.6; − 4.1–2.9; *p* = .741), non-advanced and no dementia (− 1.5; − 5.2–2.2; *p* = 0.428), or advanced and non-advanced dementia (0.9; − 2.8–4.6; *p* = .632) (Table 2). The baseline-adjusted mean differences in quality of care and dying scores were 2.7 in non-advanced dementia to 4.2 in no dementia (Table 3).

## Discussion

This subgroup analysis showed that the effects of PACE Steps to Success on comfort in the last week of life and on quality of care and dying in the last month of life did not differ between residents with advanced, non-advanced and no dementia.

Using a subgroup analysis of a large pragmatic cluster-RCT, this study offers insight on the effects of a generalist, non-disease-specific palliative care training program designed to train nursing home staff to deliver high-quality palliative care to nursing home residents with dementia (advanced and non-advanced) and without dementia [22]. We also included a large number of residents for whom the severity of dementia was determined using validated instruments. Further, following the formal rules for planning and analysis of subgroup analysis, this subgroup analysis was pre-planned and used statistical tests of interactions, which enhance the validity of study results [21]. Nonetheless, because power calculation was not conducted for this subgroup analysis, our study might not have detected potentially important but small subgroup difference in program effects. For instance, although we found a 2.7 CAD-EOLD score point difference between residents with non-advanced and no dementia, which is close to what we considered as a clinically-important effect (i.e. CAD-EOLD score of 3 points) [4, 5], the limited power might not have allowed us to detect statistically significant differences. In addition, as the presence of dementia relied on the estimation of the staff or the GP, there might be some misclassifications, particularly among residents with difficult-to-observe mild dementia symptoms. Finally, since data were collected after death, there might also be some recall bias [4, 5].

Contrary to our hypothesis, this study showed that the effects of the program did not differ between residents with advanced, non-advanced and no dementia. For the primary outcome – comfort in the last week of life – it did not achieve better outcomes for residents without dementia or with non-advanced dementia than for those with advanced dementia. Hence, as was clear from the primary trial analyses [5], the stepwise training of nursing home staff over a one-year period was not sufficient to improve comfort in the final days of life, which might be related to the intervention itself, the quality of its implementation in several nursing homes, a possible mismatch between the intervention and the primary outcome, or a combination of these factors [5, 23]. For instance, PACE Steps to Success was fully implemented as intended only in 28 of the 37 intervention nursing homes in terms of the number, order and timing of training sessions; and all 6 steps were taught in the right order and within 8 months. In seven other nursing homes, the six steps were taught but not in the right order and/or not within 8 months. In two nursing homes, they only completed five steps. Further, the adoption rates for the program materials (e.g. advance care planning material for residents) varied between countries but also fluctuated within countries [23]. While PACE Steps to Success might have addressed essential domains of palliative care that have been widely recommended for residents with and without dementia (i.e. person-centered care, advance care planning, optimal symptom assessment and management until the end of life, education of and support for healthcare providers, and support for family) [24–26], the sub-optimal implementation of the program in several nursing homes might have attenuated its effects on residents’ comfort at the end-of-life [23].

Regarding the secondary outcome, the PACE program appeared to improve quality of care and dying in the last month of life equally for those with dementia (regardless of the stage) and those without dementia. Although these findings need to be interpreted cautiously as this is a secondary outcome, they are remarkable, as this palliative care program only had a limited number of dementia-specific elements as part of the training [4]. However, the Quality of Dying in Long Term Care (QOD-LTC) scale individual items that differed between the intervention and control groups included ‘receiving affectionate touch’, ‘keeping clothes and body clean’, ‘residents appearing to be at peace’, ‘being prepared to die’, and ‘maintaining their sense of humor’. [5] Such quality of care and dying topics are not directly related to the cognitive, functional or other specific problems in dying nursing home residents with dementia [9, 27], which might explain why the effects did not differ between the subgroups.

Overall, our study implies that such a generalist and non-disease-specific palliative care program for nursing homes has the potential to improve quality of care and dying in the last month of life for both residents with and without dementia, though this finding requires further investigation and effects were only medium-sized [5]. Nevertheless, our study provides crucial insight for future developers of palliative care programs aiming to improve quality of life and dying of nursing home residents with and without dementia. A broad palliative care training program, such as PACE Steps to Success, can be a useful starting point for further improvement in palliative care in nursing homes. However, as in the primary trial analyses [5], this subgroup analysis emphasizes that this program needs to be developed further for both residents with and without dementia, e.g. to effectively promote comfort in the last days of life, either in terms of its components or the implementation processes in practice [26, 28]. Especially for dementia, as end-of-life symptoms might be very specific compared with other diseases, a strong collaboration among experts in research and practice in palliative care and dementia seems important [11, 26]. Future research evaluating palliative care programs should take into account dementia as an important subgroup, as prevalence is high in all countries, and nursing home residents die at varying stages of dementia [3].

## Conclusion

This subgroup analysis showed that the effects of PACE Steps to Success did not differ between residents with advanced, non-advanced and no dementia. These findings suggest that this program did not improve comfort in the last week of life for residents with or without dementia, but it appeared to improve quality of care and dying in the last month of life equally for residents with dementia (regardless of the stage) and without dementia. A generalist and non-disease-specific palliative care training program, such as PACE Steps to Success, can be a useful starting point for future development of palliative care programs in nursing homes. However, PACE Steps to Success needs to be developed further, so that it can effectively improve the quality of life and dying of both residents with and without dementia, e.g. by integrating components to improve residents’ comfort at the end of life.

## Supplementary Information

**Additional file 1.**

**Additional file 2.**

## Data Availability

The data that support the findings of this study are available upon request by e-mail to the project coordinator of PACE. The data can be accessed by researchers whose proposed use of the data for research purposes has been approved by the PACE consortium.

## Abbreviations

ADL: Activities of Daily Living
BANS-S: Bedford Alzheimer Nursing Severity-Scale
CAD-EOLD: Comfort Assessment in Dying–End-of-Life in Dementia
CI: Confidence interval
CPS: Cognitive Performance Scale
QOD-LTC: Quality of Dying in Long Term Care
GDS: Global Deterioration Scale
GP: General practitioners
RCT: Randomized controlled trial
