# Supplementary material for: No difference in effects of ‘PACE steps to success’ palliative care program for nursing home residents with and without dementia: a pre-planned subgroup analysis of the seven-country PACE trial
Source: BMC Palliat Care. 2021 Mar 7;20:39. doi: 10.1186/s12904-021-00734-1 (PMC7937240; doi:10.1186/s12904-021-00734-1)
Supplement: Supplementary file 1 — Additional file 1. [file 12904_2021_734_MOESM1_ESM.pdf]

PACE- comparing the effectiveness of PAlliative CarE for older people in long term care facilities in Europe. Grant agreement No 603111

Deliverable 6.3 Data analyses plan study II including cost-effectiveness analysis

Outcome, process, and cost-effectiveness evaluation of 'PACE Steps to Success' palliative care programme in long-term care facilities in Europe
Cluster-randomised controlled trial (PACE Study II)
31 January 2017

Outcome evaluation plan prepared by VUB
Process evaluation plan prepared by VUmc
Cost-effectiveness evaluation plan prepared by Radboud UMC

Overall version prepared by WP6 Lead VUB

Evaluation plans

1. **Outcome evaluation plan**
2. **Process evaluation plan**
3. **Cost-effectiveness evaluation plan**

The PACE project has received funding from the European Union's Seventh Framework Programme for research, technological development and demonstration under grant agreement no 603111.

I. Outcome evaluation plan – prepared by the VUB

Section I contains the outcome evaluation plan. This section includes the statistical analysis plan to evaluate the outcome of 'PACE Steps to Success' palliative care programme as described in WP3 of the Annex I of the Grant Agreement and to assess whether or not outcomes vary between different sub-groups. Figure 1 illustrates the data collection methodology used for the outcome evaluation.

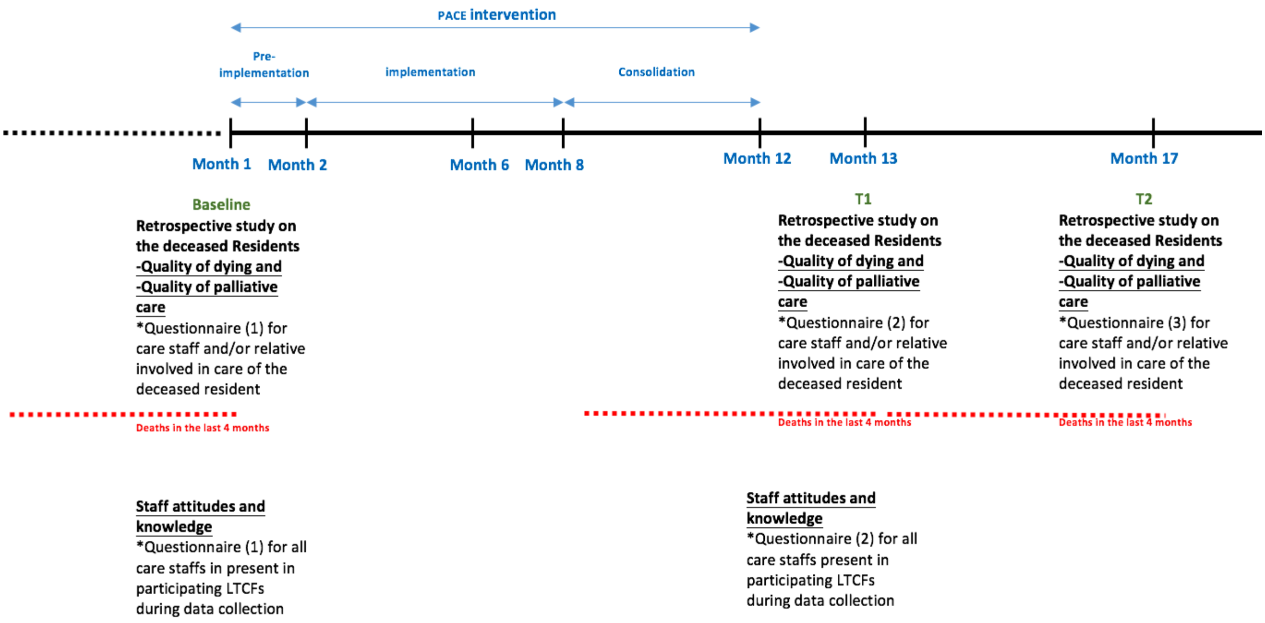

Figure 1. Data collection methodology for the outcome evaluation of PACE

STATISTICAL ANALYSIS PLAN

Hypotheses for primary outcome measures

Hypothesis 1: Quality of dying of residents as evaluated by staff is better in facilities using the 'PACE Steps to Success' palliative care programme than facilities which provide care as usual compared to baseline.

Hypothesis 2: Staff attitudes and knowledge towards palliative care are better in facilities using the 'PACE Steps to Success' palliative care programme than facilities which provide care as usual compared to baseline.

Hypotheses for secondary outcome measures

Hypothesis 3: Quality of end-of-life care as evaluated by relatives is better in facilities using the 'PACE Steps to Success' palliative care programme than facilities which provide care as usual compared to baseline.

Primary outcome measures

Hypothesis 1: Quality of dying of residents

The outcome measures to test hypotheses 1 include several aspects of quality of dying of the residents of the participating long-term care facilities (LTCFs).

- End-of-Life in Dementia – Comfort Assessment in Dying (EOLD – CAD)
    - Comfort during the last 7 days of life
    - Answered by the staff most involved in care and the most closely involved relative of the deceased resident at baseline, month 13 (T1), and 17 (T2)
- Quality of Dying in Long-Term Care (QOD – LTC):
    - Quality of dying in long-term care
    - Answered by the staff most involved in care for the deceased resident at baseline, month 13 (T1) and 17 (T2)

Hypothesis 2: Staff attitudes and knowledge towards palliative care

The outcome measures to test hypotheses 2 include several aspects of the attitudes and knowledge towards palliative care of staff in the participating LTCFs. These outcome measures were answered by all care staff present in participating LTCFs during data collection at baseline and month 12.

- End-of-Life Professional Caregiver Survey (ECPS)
    - Staff attitudes towards patient and family communication, cultural, and ethical values
- Palliative Care Survey (PCS)
    - Staff knowledge on palliative care
- Self-Efficacy in End-of-Life Care Survey (S-EOLC)
    - Staff confidence in communicating with residents and families about end-of-life (i.e. self-efficacy)

Secondary outcome measures

Hypotheses 3*:* Quality of end-of-life care

The outcome measure to test hypotheses 3 includes an aspect of the quality of end-of-life care.

- End-of-Life in Dementia – Satisfaction with Care (EOLD – SWC)
    - Evaluation of the quality of end-of-life care
    - Answered by the most closely involved relative of the deceased resident at baseline, month 13 (T1) and 17 (T2).

Sample

Hypothesis 1: To test hypotheses 1, all deceased residents for whom the staff most involved in care for the deceased resident completed a questionnaire will be included in the sample.

Hypothesis 2: To test hypotheses 2, all care staff present in participating LTCFs during data collection, who completed a questionnaire regarding their attitudes and knowledge towards palliative care, will be included in the sample.

Hypothesis 3: To test hypotheses 3, all deceased residents for whom the most closely involved relative of the deceased resident completed a questionnaire will be included in the sample.

Data analyses

Step 1. Exploring the data and data assumptions

The nature of each variable, as well as the influence of certain data assumptions on the study results must be explored prior to primary data analyses.

Step 1.1. Explore the number of cases in T1 and T2 to determine statistical power for each post intervention measurements. T1 and T2 can be used separately for the analysis when statistical power is at least 80%.

Step 1.2. Due to potential turn-over of staffs in LTCFs, the samples – care staffs present in participating LTCFs during data collection– with both the baseline and post-intervention measurements will be explored.

Step 1.3. Check data assumptions for regression analyses.

Normality of distribution – For continuous variables, a graphical representation (histogram) or the Shapiro Wilk-test or Kolmogorov Smirnov test can be used to check for normality of the distribution. If the normality of distribution is violated, log-transformation may for example be done. The partner responsible for conducting the analysis would need to discuss in the consortium on how skewed data can further be used for the analysis.

Linearity – Linearity between the outcome measures and the intervention variable or covariates will be checked through visual inspection of the scatter diagram. If the assumption of linearity is violated, the partner responsible for conducting the analysis would need to discuss in the consortium on how these data can be further used for the analysis – e.g. transforming into quartiles.

Step 1.4. Prepare the variables to be used for the data analyses based on the exploration of data and data assumptions.

Step 2. Dealing with missing values

The PACE Study II methodology and data management have been meticulously designed. Hence, when dealing with missing values, the basic assumption is that data are missing completely at random and non-response is unrelated to any particular group or the actual values of the missing data. Nevertheless, this assumption will still be examined prior to data analysis through the following steps:

Step 2.1. Analyse the type of missing values, if known – e.g. refusal or coding error.

Step 2.2. Compare the sample of respondents with and without missing values to check for potential variations.

Step 2.3. Compare respondents and non-respondents to check for potential variations. This can be

done by using the information given by other respondents regarding the same deceased resident.

To handle missing values, multiple imputation – i.e. a simulation-based approach that takes the uncertainty of the imputed values into account – can be used. With multiple imputation, cases with missing values will still be included in the sample in order to manage potential bias due to non-random deletion of cases.

Each outcome variable will be constructed by summing the value of multiple items and thus missing values may also occur within a certain outcome variable. This study allows missing items not exceeding 30% of the total number of items for each outcome variable. For example, EOLD-CAD is the sum score of 14 symptoms and conditions assessed from a scale of 1 to 3. Only EOLD-CAD data with not more than 4 missing items will be included in the analysis. For EOLD-CAD data with missing items, the sum score will be calculated with a different denominator. This procedure also applies to other outcome variables in the study. The potential impact of missing values on the findings of the study will also be addressed in the Discussion section of papers written with PACE Study II data and the final report of study II.

Step 3. Descriptive statistics

The characteristics of the deceased residents will be described in terms of age, gender, socio-economic status, cause of death or disease underlying death, and functional and cognitive status. The characteristics of the care staff present in participating LTCFs during data collection will be described in terms of age, gender, experience with care, level of education, and palliative care training.

Baseline and post-intervention measurement characteristics will be summarized, both for the control group and the intervention group. The mean and standard deviation or proportion will be given for the descriptive variables. Anova (normal distribution) or Mann-Whitney U-test (non-normal distribution) for continuous, and χ^2 tests for categorical variables will be used to describe differences between the control group and the intervention group in the baseline and post-intervention measurements and for non-response analysis.

A visual representation of differences in the characteristics of the sample between the control group and the intervention group and between baseline and post-intervention measurements may also be provided by using graphs or plots.

Step 4. Conducting statistical analyses to test study hypotheses

Intention-to-treat and per protocol analysis

The primary statistical analyses will use the intention-to-treat (ITT) approach. With the ITT approach, the outcome data from all of the samples who were enrolled and randomized to the intervention group or the control group will be accounted for in the main analyses in the original groups to which they were randomized, regardless of whether or not they completed the PACE Steps to Success training. This approach may reflect the

effects of ‘PACE Steps to Success’ palliative programme on quality of dying of residents, staff attitudes and knowledge towards palliative care, and quality of palliative care in daily practice.

Per protocol analysis will also be performed. This includes LTCFs which were able to complete the study “according to the protocol”. Based on the process evaluation measures, we will determine the cut offs to identify which LTCFs had followed the ‘PACE Steps to Success’ palliative care programme protocol. Per protocol approach may lower evidence level by introducing attrition bias because those who were only able to attend 2 of the 6 PACE trainings for instance may have different characteristics than those who attended all training sessions. Nonetheless, this approach better reflects the effects of ‘PACE Steps to Success’ palliative programme on quality of dying of residents, staff attitudes and knowledge towards palliative care, and quality of palliative care when intervention is implemented and taken in an optimal manner.

Regression analyses

To test the study hypotheses, several regression analyses will be conducted based on the exploration of data and data assumptions, as well as on the data analysis approach to be used (ITT and per protocol). Generally, all primary and secondary outcome measures (scale sum scores) are by nature continuous variables, which would require multivariate linear regression analysis techniques. However, if the assumptions of normal distribution and/or linearity is/are violated, multivariate logistic (or ordinal) regression analyses may be applicable. The specific procedure on how to further analyse skewed or non-linear data will rely upon the decision of the consortium. Basically, multivariate linear or logistic regression analyses may be performed to assess and compare the baseline and post-intervention measurement differences in the outcome measures between the intervention group and the control group, while adjusting for sample characteristics that may differ between groups.

Additionally, multi-level mixed model regression analyses will be performed to account for the baseline measurement and the multilevel nature of the data – e.g. residents and staff nested within LTCFs or country. The kind of multilevel mixed model regression analyses will depend on the nature of the outcome variables and the statistical package to be used for the main analyses.

With the multi-level mixed model analyses, outcomes will be analysed with LTCFs and country as random factor, and group, time point, and their interaction as fixed factors. Differences in mean change (post-intervention measurements minus baseline) between the intervention group and the control group (interaction group*time) will be calculated. Estimated means with corresponding 95% CI will be reported at baseline and follow-up, both for the intervention and control group. Moreover, estimated differences (and 95%CI) in change between intervention and control are reported. The effect sizes (Cohen’s *d*) using the baseline-adjusted mean differences and the variance between residents or care staffs, between LTCFs, and between country will be estimated to assess the magnitude of the effects for the different outcome measures.

All analyses will be two-tailed and considered significant if $\alpha = 0.05$. In addition, data will be analysed by using statistical software program suitable for the necessary statistical analyses, such as multilevel mixed model analysis and multiple imputation – e.g. STATA, SAS, or IBM SPSS.

SUB-ANALYSES

The primary aim of the sub-analyses is to evaluate the effectiveness of the 'PACE Steps to Success' palliative care programme on different subgroups. For instance, the sub-analysis may aim to elucidate whether the effects of the 'PACE Steps to Success' palliative care programme on quality of dying of residents vary by characteristics of the deceased resident (i.e. age and gender, socio-economic status, clinical characteristics – e.g. cognitive status – dementia diagnosis –, and functional status). Each partner organization will create their own sub-analysis plan relevant to their research questions and hypotheses.

An example of a sub-group analysis

To demonstrate how a sub-analysis can be performed, a concrete example is provided.

Research aim: In this example, the sub-analysis aims to assess whether the outcomes of the 'PACE Steps to Success' palliative care programme on quality of dying differ between residents with and without dementia.

SUB-GROUP DATA ANALYSIS

For information regarding the **OUTCOME MEAUSURES**, **SAMPLE**, **EXPLORATION OF DATA AND DATA ASSUMPTIONS**, **PROCEDURES TO HANDLE MISSING VALUES**, and **DATA ANALYSIS APPROACH** (ITT and per protocol analysis), please refer to the main STATISTICAL ANALYSIS PLAN. Below the specifics for the sub-analyses are described.

Descriptive statistics

The characteristics of the deceased residents with and without dementia in facilities using the 'PACE Steps to Success' palliative care programme and in facilities which provide care as usual will be provided in terms of age, gender, socio-economic status, cause of death or disease underlying death, and functional and cognitive status.

Baseline and post-intervention measurement characteristics will be summarized separately for residents with and without dementia, both for facilities using the 'PACE Steps to Success' palliative care programme and facilities which provide care as usual. The mean and standard deviation or proportion will be given for the descriptive variables. Anova (normal distribution) or Mann-Whitney U-test (non-normal distribution) for continuous, and χ^2 tests for categorical variables will be used to assess differences between residents with and

without dementia in the baseline and post-intervention measurements and for non-response analysis, both for the intervention group and the control group.

A visual representation of differences in the characteristics of the samples between residents with and without dementia, both for the control group and the intervention group, and between baseline and post-intervention measurements may also be provided by using graphs or plots.

Regression analyses

For this sub-analysis, several regression analyses will be conducted based on the exploration of data and data assumptions, as well as on the data analysis approach to be used (ITT and per protocol). Generally, the EOLD-CAD or QOD-LTC variables are by nature continuous variables, which would require multivariate linear regression analysis techniques. However, if the assumptions of normal distribution and/or linearity is/are violated, multivariate logistic regression analyses may be applicable. The specific procedure on how to further analyse skewed or non-linear data will rely upon the decision of the consortium. Basically, multivariate linear or logistic regression analyses may be performed to assess and compare the baseline and post-intervention measurement differences in the quality of dying between residents with and without dementia, both for the intervention group and the control group, while adjusting for sample characteristics that may differ between groups.

Additionally, multi-level mixed model regression analyses will be performed to account for the baseline measurement and the multilevel nature of the data – e.g. residents and staff nested within LTCFs or country. The kind of multilevel mixed model regression analyses will depend on the nature of the outcome variables and the statistical package to be used for the main analyses.

With the multi-level mixed model analyses, the outcomes will be analysed with LTCFs and country as random factor, and group, time point, and their interaction as fixed factors. Differences in mean change (post-intervention measurements minus baseline) between deceased residents with and without dementia, both for the intervention group and the control group (interaction group*time*dementia) will be calculated. Estimated means with corresponding 95% CI will be reported at baseline and follow-up, both for the intervention and control group. Moreover, estimated differences (and 95%CI) in change between intervention and control will be reported. The effect sizes (Cohen's *d*) using the baseline-adjusted mean differences and the variance between residents or care staffs, between LTCFs, and between country will be estimated to assess the magnitude of the effects for the different outcome measures.

All analyses will be two-tailed and considered significant if $\alpha = 0.05$. In addition, data will be analysed by using statistical software program suitable for the necessary statistical analyses, such as multilevel mixed model analysis and multiple imputation – e.g. STATA, SAS, or IBM SPSS.

II. Process evaluation plan – prepared by VUmc

Section II contains the process evaluation plan. This section includes the plan to evaluate the implementation process of the 'PACE Steps to Success' palliative care programme in long-term care facilities and to identify facilitators and barriers across countries and specific countries.

MEASURES

The process evaluation follows the RE-AIM framework to structure the different implementation factors that are considered important for implementation effectiveness, namely Reach, Efficacy, Adoption, Implementation, and Maintenance (Glasgow *et al.*, 1999)[1]. An overview of the measures used in the process evaluation can be seen in Table 1.

Table 1. Operationalization of RE-AIM dimensions and measurement methods

RE-AIM EVALUATION DIMENSIONS		
**DIMENSIONS**	**OPERATIONALIZED IN PACE PROCESS EVALUATION**	**MEASUREMENT METHODS**
Reach	Number of participants (care staff attending each training or meeting) divided by the total number of care staff (eligible participants) who work on the LTCF or LTCF unit<br><br>Comparing characteristics of participating LTCFs with non-participating LTCFs	Attendance lists<br><br>Documentation of recruitment process
Efficacy	Primary and secondary outcome measures (as described in section I)	Questionnaires (see section I)
Adoption	Adherence: trend in attendance to training session 1 up to training session 6<br><br>Number of Looking and Thinking Ahead Forms documented and Pain/Depression Assessments documented<br><br>Experiences with applying the intervention steps in daily practice (e.g. reasons for (not) applying steps, changes in practice)	Attendance lists<br><br>Report from PACE coordinators at month 12<br><br>Group interviews with care staff and PACE coordinators

[1] Glasgow RE, Vogt TM, Boles SM: Evaluating the public health impact of health promotion interventions: the RE-AIM framework. Am J Public Health 1999, 89: 1322-1327.

Table 1 continued. **Operationalization of RE-AIM dimensions and measurement methods**

RE-AIM EVALUATION DIMENSIONS continued		
**DIMENSIONS**	**OPERATIONALIZED IN PACE PROCESS EVALUATION**	**MEASUREMENT METHODS**
Implementation	Fidelity: extent to which the steps of the intervention were delivered as intended (frequency, order and content of the sessions)<br><br>Consistency of implementation across LTCF-settings	Structured diaries for country trainers
	Satisfaction of care staff members towards the intervention program, trainer's competences and number of coaching contacts	Evaluation questionnaire after last training session (month 8)
	Barriers and facilitators for implementation	List of Barriers and Facilitators for Implementation, added to the Nurses' experiences and attitudes questionnaire at month 13 (T1)<br><br>Group interviews with care staff and PACE coordinators<br><br>Online discussion groups with trainers from all countries<br><br>Semi-structured interviews with facility managers<br>Structured diaries for country trainers and PACE coordinators
Maintenance	Care staff members' intention for using PACE documents in the future	Evaluation questionnaire after last training session (month 8)
	Organizational intention for long-term implementation	Semi-structured interviews with facility managers
	Recommendations for improving usability of intervention program	Group interviews with care staff<br>Group interview with PACE coordinators<br>Online discussion group with trainers from all countries

DATA ANALYSIS FOR QUANTITATIVE MEASUREMENTS

We will calculate descriptive statistics for the following quantitative measures:

<u>1. Documentation of recruitment process</u>

RE-AIM element: Reach

Each country has documented the way they recruited LTCFs to participate in the PACE project. In this documentation, information is gathered on the characteristics of participants vs. non-participants.

Per country, we will calculate:

- The percentage of private and public LTCFs among participating LTCFs and non-participating LTCFs.
- The mean number (and range) of beds among participating LTCFs and non-participating LTCFs (if possible; some countries approached LTC organizations instead of individual LTCFs).

2. Attendance lists for training sessions, multidisciplinary meetings and reflective debriefing sessions

RE-AIM elements: Reach, Adoption

Per training session, multidisciplinary meeting and reflective debriefing session, PACE coordinators registered the attendance of LTCF staff members. Attendance lists were filled in on paper (except in one country where it was filled in digitally), and later entered in a database. They contain the following information:

- Total number of care staff at unit(s) were PACE is implemented (at time of training/meeting)
- Number of care staff that attended the training/meeting
- Number of care staff members with the following reasons for not attending the training/meeting
  - Not working today
  - Sick leave
  - Holiday
  - On duty on ward
  - Other
- Number of other professionals attending the multidisciplinary meeting or reflective debriefing session

Outcome variables:

Per LTCF and per training session, multidisciplinary meeting or reflective debriefing session will be described:

- Number of care staff attending each session or meeting divided by the total number of care staff (eligible participants) who work on the LTCF or LTCF unit
- Whether or not other professionals than care staff members were involved in multidisciplinary meetings or reflective debriefing sessions
- Frequency of most important reasons for not attending training/meeting (and thus affecting the reach of the intervention).

Per LTCF we will then calculate:

- Mean percentage of care staff members attending the training sessions
- Mean percentage of staff members attending multidisciplinary meetings
- Mean percentage of staff members attending reflective debriefing sessions

We will also describe the trend in attendance to training session 1 up to training session 6.

3. Diaries for Country Trainers

RE-AIM element: Implementation

During 12 months (pre-phase, intervention phase and consolidation phase), Country Trainers have registered all activities they performed with regard to the PACE Program. They updated their digital diary on a weekly basis. Their diaries contain information on the following variables:

Per week:

- Time spent on preparation for training sessions
- Time spent on other overarching activities not bound to a specific LTCF (+ details on these activities)

Per week and per LTCF:

- Time spent on providing training sessions
- Time spent on visits to care home (for another reason than delivering a training)
- Time spent on contacts via email/telephone
- Time spent on other activities (+ details on these activities)

Outcome variables:

Per trainer, we will calculate the total time over 12 months as well as the average time per week spent on:

- Preparation for training sessions
- Other overarching activities not bound to a specific LTCF
- Providing training sessions
- Visits to care home
- Contacts via email/telephone
- Other activities
- PACE (in total – all activities together)

These outcome variables will be adjusted for the number of LTCFs that a country trainer was responsible for.

In addition, we will extract per LTCF whether the training program has been delivered as intended (fidelity) with regard to frequency, order and content of steps (all steps 1 to 6 delivered in the appropriate order and one training session each month).

4. Diaries for PACE coordinators

RE-AIM element: Implementation

During 18 months (pre-phase, intervention phase, consolidation phase plus 6 months extra), PACE coordinators have registered all activities they performed with regard to the PACE Program. Diaries were filled in on paper

(except in one country where it was digital) on a weekly basis, and later entered in a database. Their diaries contain information on the following variables:

Per week:

- Time spent on PACE activities
- Type of activity performed (with answer options: attending one of the six PACE training sessions, preparation of multidisciplinary meeting, leading or attending a multidisciplinary meeting, preparation of reflective debriefing session, leading or attending a reflective debriefing session, (telephone) meeting with PACE country trainer, other (telephone) meeting regarding PACE, other)

Outcome variables:

Per PACE coordinator, we will calculate

- the total time over 18 months as well as the average time per week spent on PACE activities
- the number of weeks in which PACE coordinators
    - attended one of the six training sessions
    - prepared a multidisciplinary meeting
    - leaded or attended a multidisciplinary meeting
    - prepared a reflective debriefing session
    - leaded or attended a reflective debriefing sessions
    - had a (telephone) meeting with PACE country trainer
    - had another meeting regarding PACE
    - did other PACE activities

These outcome variables will be adjusted for the number of PACE coordinators that were appointed in the LTCF.

5. Evaluation questionnaire after 6th training session

RE-AIM elements: Implementation, Maintenance

After the last training session (step 6), all care staff members received a written evaluation questionnaire. The evaluation questionnaire is linked to the same staff member as in the baseline measurement (T0), by using the same list linking the anonymous code to the listed staff member (kept in the LTCF). In this way, it can be investigated whether the number of sessions that an individual care staff member reported to have attended is associated with the differences found between measurements T0 and T1. Information from the evaluation questionnaires will be entered in a database (LimeSurvey).

The evaluation questionnaires contain information on the following variables:

- Characteristics of participants (age, gender, function, number of hours per week working in LTCF)

- Satisfaction with trainer (regarding 4 aspects – expertise, conveying theory and skills, giving room for questions and discussions, overall teaching competences)
- Evaluation of number and length of training sessions
- Which training sessions were attended
- Evaluation of how much was learned from each training session
- Evaluation of how difficult or easy it was to fill in PACE documents
- Evaluation of usefulness of PACE documents
- Intention to use PACE documents in the future
- Overall evaluation of complete PACE Program
- Confidence towards PACE Program supporting staff to provide good palliative care
- Whether or not the PACE Program would be recommended to other LTCFs

6. Report with number of PACE documents filled in at month 12

RE-AIM element: Adoption

Per LTCF, PACE coordinators registered the following information at month 12:

- Number of residents with/for whom a Looking and Thinking Ahead document is filled out
- Number of residents with/for whom (at least once) a Cornell Depression Scale has been filled out
- Number of residents with/for whom (at least once) a Geriatric Depression Form has been filled out
- Number of residents with/for whom (at least once) LTCF Pain assessment has been filled out
- Number of residents with/for whom (at least once) PAINAD has been filled out
- Total number of beds on PACE wards

Outcome variables:

Per LTCF and per document or measurement scale, we will calculate:

- The number of residents with/for whom a document/scale has been filled out divided by the total number of beds on PACE wards

7. List of barriers and facilitators in T1 measurement

RE-AIM element: Implementation

In the T1 measurement questionnaire for care staff members of intervention LTCFs (distributed at month 13), 8 statements on barriers and facilitators for implementing the PACE Program were formulated. Care staff members could indicate to what extent they agreed with each statement on a 5-point scale (1 = fully disagree, 5 = fully agree).

In order to identify barriers for implementation, the percentage of care staff members that agree or fully agree with the following negatively formulated items will be calculated per intervention LTCF:

- Working on the PACE programme is time consuming
- Fellow nursing staff members do not apply the PACE programme
- I have problems changing my old routines
- I have a general resistance to working with protocols

Also the percentage of care staff members that disagree or fully disagree with the following positively formulated items will be calculated per intervention LTCF:

- Working on the PACE programme has improved the quality of care on the ward
- Working on the PACE programme has increased my job satisfaction
- Working on the PACE programme has been rewarding to me
- Working on the PACE programme has more benefits than burdens to me

DATA ANALYSIS FOR QUALITATIVE MEASURES

For the qualitative measures, thematic content analyses will be used, using codes on the basis of the underlying structure of the interviews and open-ended questions.

1. Group interviews with care staff, PACE coordinators or semi-structured interviews with facility managers

RE-AIM elements: Adoption, Implementation, Maintenance

These interviews will be analyzed by undertaking the following steps:

a) Directly after the interview a draft summary will be written in English, using a template that follows the main topics from the interview topic list. This summary is based on the short-term memory of the interviewer. This summary will be perfected/completed at a later moment.
b) The interview will be transcribed in the native language.
c) The transcript will be analyzed according to the principles of thematic analysis. In thematic analysis, themes or patterns within data can be identified in one of two primary ways: in an inductive or 'bottom up' way, or in a theoretical or deductive or 'top down' way (also known as 'framework approach'). We will do it in a more theoretical/deductive top down way, meaning that we already chose to pre-structure the themes in the templates for summaries.
d) Based on the analysis of transcripts, the draft summary will be adjusted, perfected and completed. Also interesting and illustrating quotes will be added to the summary.
e) Summary and quotes will be checked by another researcher from the same country. This means that another researcher first reads the entire transcript, then checks whether the ascribed codes are accurate and complete, and also reads and checks the summary.

f) Each summary will be sent to the coordinator, who collects all summaries, reads them for initial understanding, may ask for clarification or more quotes, and will distribute (some of) them to all researcher who will attend the Google Hangout Meetings.

g) Researchers from all countries read these (synthesis of) summaries and have regular Google Hangout meetings in which experiences will be discussed and findings will be analyzed through an interactive ongoing process of discussion.

h) When all interviews of one specific type (e.g. interviews with facility managers) have been completed in one country, an overall written English summary of the data of these interviews will be written using a template.

i) Researchers from all countries further analyze the cross country data together, facilitated by summaries and Google Hangout meetings. The result of this step will be a report/article(s).

2. Online group discussions with country trainers

RE-AIM elements: Implementation, Maintenance

Transcripts of the online group discussions with country trainers are immediately available and already in English. They will be analyzed following the principles of thematic analysis – in a theoretical/deductive 'top down' way, meaning that we already chose to pre-structure the themes to some extent. As a first step of analysis transcripts will be read and reread to allow the researchers to become thoroughly familiar with the data. Codes will then be ascribed to meaningful text units and grouped together. The findings will be discussed with different members of the research team, in order to work toward a consensus about interpretation of the key findings.

3. Structured diaries for country trainers and PACE coordinators

RE-AIM element: Implementation

In addition to quantitative information, the diaries for country trainers and PACE coordinators contain open-ended questions on successes and challenges that they encounter during PACE activities.

Per LTCF, successes as well as challenges will be analyzed by ascribing codes to the answers of PACE coordinators and country trainers. Then, these codes will be grouped together. The findings will be discussed with different members of the research team, in order to work toward consensus about interpretation of the key findings.

COMBINING RE-AIM ELEMENTS

There is no validated strategy to combine RE-AIM elements into one overall score. One approach to combine the different RE-AIM elements is to visually display how each LTCF within each country scores on each RE-AIM

dimension. Based on the key elements of the PACE Program and each RE-AIM dimension, and based on the (range of) descriptive statistics and other outcomes that we will gather with above described measurement methods, we will define the criteria for a low, medium and high level of Reach, Adoption, Efficacy, Implementation and Maintenance. We can visually display how each LTCF within each country performed on the different dimensions. In this way, the strengths and limitations of implementing the PACE Program in different LTCFs and countries can quickly be seen (see Figure 2 for an example).

Figure 2. Example of visually displaying RE-AIM dimensions.

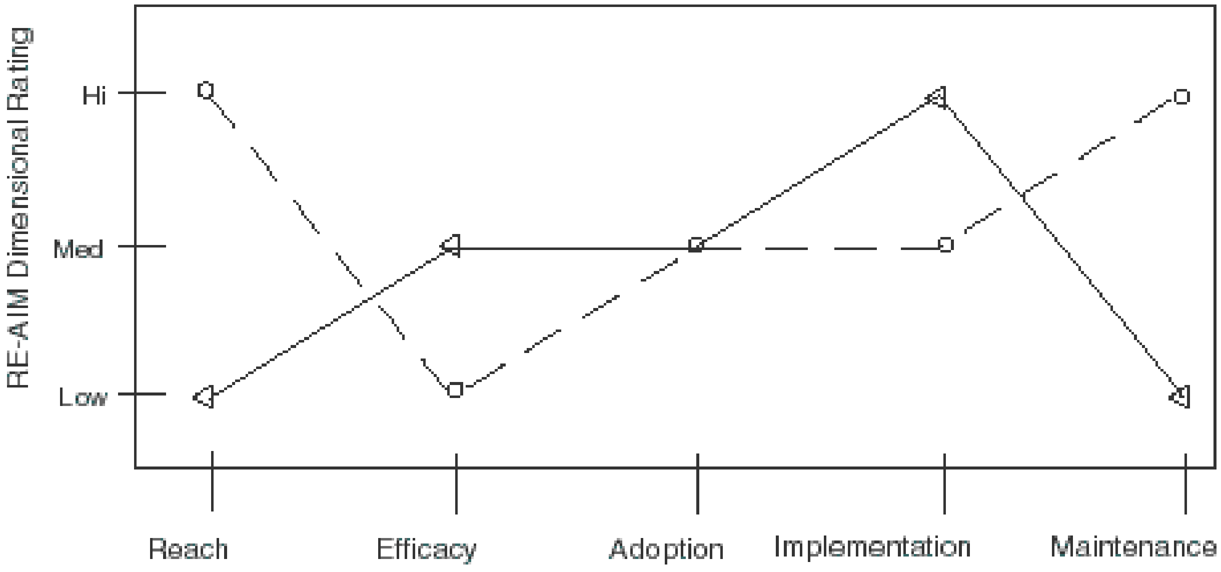

III. Cost-effectiveness evaluation plan – prepared by Radboud UMC

Section III contains the cost-effectiveness analysis plan. This section includes the plan on how to evaluate the cost-effectiveness of the 'PACE Steps to Success' palliative care programme in comparison to end-of-life care provided as usual.

DATA COLLECTION

Data were collected within the PACE Study II project. Retrospective data collection of deceased residents took place at the start of the trial (month 1) and at month 13 and 17 of the trial.

PACE staff questionnaires regarding care services received during the *last month of life* are used to determine resource use by residents from. Direct medical resource use was collected on a per patient basis in the participating facilities, using (parts of) the resource use in dementia (RUD). Direct medical costs include care received in the last months of life, such as hospital admission, care received in an emergency room, treatments in the last month of life, adverse events in the last month of life, or opioids, antipsychotics, hypnotics or sedatives. Additionally, data on PACE-related costs are also collected.

Calculating costs

The cost to be calculated will include direct medical costs and the PACE-related costs. Cost calculation will be the product of volumes of resource use multiplied by prices. After resources use data were gathered, costs were estimated by multiplying quantities of resources used with standard unit cost prices in Euros, based on Dutch prices. Maximum tariffs for unit costs as set by the Dutch Health Care Authority (NZA) were used where possible. If no maximum rates are specified, 'passers-by' hospital tariffs were used. The price vector will be adjusted for differences in price levels across countries, using the purchasing power parity (PPP) technique.

DATA ANALYSIS

A resident-based economic evaluation based on the general principles of a cost-effectiveness (utility) analysis was performed to analyze the cost-effectiveness of the 'PACE 6 Steps to Success' palliative care programme in comparison to palliative care provided as usual.

Outcome measures

Primary outcome measures for the economic evaluation, considering last month before dying, are direct costs and quality-adjusted life years (QALYs), which will be based on scores on the EuroQol-5D (EQ5D-5L) as well as the Quality of Dying in Long-term Care (QOD-LTC) measure.

Incremental cost-effectiveness ratio

Incremental cost-effectiveness rations (ICERs) 'cost per quality increase based on EQ5D-5L utilities' as well as 'cost per quality increase as measured by the QOD-LTC' will be computed. Uncertainty surrounding the ICERs will be determined by using the bootstrap method or the Fieller method.

The ICER (cost per QALY gained) will be transformed in the parameter 'Net Monetary Benefit'. In a regression approach Net Monetary Benefit is the dependent variable. Independent variables are group (PACE, control), LTCF (cluster) and potential confounders. We will estimate 6 models, varying the WTP for a QALY over a range [€0-€100.000] in steps of €20.000 and present the results via a Cost-Effectiveness Acceptability Curve (CEAC). Depending on the skewness of cost (NMB with WTP=0), we will apply a generalized linear model (GLM) approach using a gamma distribution with log link function with cluster robust standard errors. For NMBs with WTP>0 we apply a GLM with log or identity link depending on skewness of the distribution, with cluster robust standard errors.

A cost-effectiveness acceptability curve will be derived that is able to evaluate efficiency by using different thresholds (willingness to pay) for a QALY. The impact of uncertainty surrounding deterministic parameters (for example prices) on the ICER will be explored using one-way sensitivity analyses on the range of extremes. Potential differences in transferability of cost and effect data between countries will be accounted for in scenario analyses.
