# Supplementary material for: No difference in effects of ‘PACE steps to success’ palliative care program for nursing home residents with and without dementia: a pre-planned subgroup analysis of the seven-country PACE trial
Source: BMC Palliat Care. 2021 Mar 7;20:39. doi: 10.1186/s12904-021-00734-1 (PMC7937240; doi:10.1186/s12904-021-00734-1)
Supplement: Supplementary file 2 — Additional file 2. [file 12904_2021_734_MOESM2_ESM.pdf]

Supplemental file 2. Comprehensive description of the primary and secondary outcomes

Comfort Assessment in Dying–End-of-Life in Dementia-scale (CAD-EOLD) scale

Comfort Assessment in Dying–End-of-Life in Dementia-scale (CAD-EOLD) scale was used to measure comfort in the last week of life. The EOLD-CAD scale comprises 14 individual items (discomfort, pain, restlessness, shortness of breath, choking, gurgling, difficulty swallowing, emotional support, fear, anxiety, crying, moaning, serenity, peace and calm) grouped into four subscales (physical distress, dying symptoms, emotional symptoms and well-being). The individual items were scored on a scale of 1 (not at all) to 3 (a lot). Items are summed to calculate an overall score between 14 and 42. The items serenity, peace and calm were reverse coded, so higher scores indicate better comfort in the last week of life.

CAD-EOLD: When you think about the resident's physical and emotional state, can you please indicate to what extent he or she experienced the following during <u>the last week of life</u>?
(*for each line, please tick a single box in the column that applies*)

	a lot	somewhat	not at all
**a.** discomfort	☐	☐	☐
**b.** pain	☐	☐	☐
**c.** restlessness	☐	☐	☐
**d.** shortness of breath	☐	☐	☐
**e.** choking	☐	☐	☐
**f.** gurgling	☐	☐	☐
**g.** difficulty swallowing	☐	☐	☐
**h.** fear	☐	☐	☐
**i.** anxiety	☐	☐	☐
**j.** crying	☐	☐	☐
**k.** moaning	☐	☐	☐
**l.** serenity	☐	☐	☐
**m.** peace	☐	☐	☐
**n.** calm	☐	☐	☐
**o.** resistiveness to care	☐	☐	☐

Quality of Dying in Long Term Care (QOD-LTC) scale

Quality of Dying in Long Term Care (QOD-LTC) scale was used to measure quality of care and dying. The QOD-LTC scale assesses perspectives on quality of personhood, closure and preparatory tasks, including 11 individual items. Items are summed to calculate an overall score between 11 and 55. Higher mean score indicates better quality of care and dying.

Please think back over the last month of life of the resident. Here are some statements that have been considered important during the last phase of life. How true is each statement for (the situation of) the resident?

(for each line, please tick a single box in the column that applies)

		**not at all**	a little	a moderate amount	quite a bit	**completely**
**A**	There was a nurse or aide with whom the resident felt comfortable	☐	☐	☐	☐	☐
**B**	The resident received affectionate touch daily	☐	☐	☐	☐	☐
**C**	The resident appeared to be at peace	☐	☐	☐	☐	☐
**D**	The resident's physician knew him or her as a whole person including life and personality	☐	☐	☐	☐	☐
**E**	The resident had treatment preferences in writing	☐	☐	☐	☐	☐
**F**	The resident indicated he or she was prepared to die	☐	☐	☐	☐	☐
**G**	The resident's funeral was planned	☐	☐	☐	☐	☐
**H**	The resident had named a decision-maker in the event that he or she was no longer able to make decisions	☐	☐	☐	☐	☐
**I**	The resident maintained his or her sense of humour	☐	☐	☐	☐	☐
**J**	The resident's dignity was maintained	☐	☐	☐	☐	☐
**K**	The resident's clothes and body were clean	☐	☐	☐	☐	☐
